# Supplementary material for: Lossy compression of matrices by black box optimisation of mixed integer nonlinear programming
Source: Sci Rep. 2022 Sep 15;12:15482. doi: 10.1038/s41598-022-19763-8 (PMC9477857; doi:10.1038/s41598-022-19763-8)
Supplement: Supplementary file 1 — Supplementary Information 1. [file 41598_2022_19763_MOESM1_ESM.pdf]

Supplementary Information for "Lossy compression of matrices by black box optimisation of mixed integer nonlinear programming."

Program code to reproduce the results are available in jupyter notebook files. There are two licenses for distributing the files; thus, we separate them into two zip files, supplementary_notebook1.zip and supplementary_notebook2.zip. Those files should be combined into the same folder to execute the program code appropriately.

The raw data from each run is not included because of storage size limitations. All program code that reproduces the raw data is provided instead. The notebooks can be executed in alphabetical order except for z007.ipynb, which summarises all the results.

Supplementary Table S1 : Contents in the supplementary file “supplementary_notebook1.zip”

File or folder(/)	Description
data/	Supplementary Data 1: Embedding data for D-Wave
results/	Supplementary Data 2: Instance data and residual error data from simulations
script/	Modules and subroutines for the main analysis
00_cpuinfo.txt	CPU information, on which the main analysis conducted
00_memo.txt	Brief description of jupyter notebooks
00_readme.txt	Direction to combine all files in two zip files
LICENSE	Licence information for the program code
Pipfile	Python package information
z000.ipynb	Notebook to generate problem instances N, D, K = 8, 100, 3
z001.ipynb	Notebook to conduct gBOCS (nBOCS) hyper parameter search<br>(sigma^2 = 0.0001, 0.001, 0.01, 0.1, 1, 10) => 0.1
z002.ipynb	Notebook to conduct sBOCS (gBOCS) hyper parameter search<br>(beta = 0.0001, 0.001, 0.01, 0.1, 1, 10, 100) => 0.001
z003.ipynb	Notebook to conduct D-wave embedding
z007.ipynb	Notebook to summarise for publication
z010.ipynb	Notebook to conduct random sampling (RS)
z015.ipynb	Notebook to conduct vanilla BOCS (horseshoe prior) (vBOCS)
z011.ipynb	Notebook to conduct BOCS with normal prior (mean) (nBOCS)
z013.ipynb	Notebook to conduct BOCS with gamma-normal prior<br>(mean + standard deviation) (gBOCS)
z016.ipynb	Notebook to conduct FMQA with k=8 (FMQA08)
z017.ipynb	Notebook to conduct FMQA with k=12 (FMQA12)
z051.ipynb	Notebook to conduct nBOCS with quantum annealing (nBOCSqa)
z019.ipynb	Notebook to conduct nBOCS with simulated quenching (nBOCSsq)
z018.ipynb	Notebook to conduct nBOCS with data augmentation (nBOCSa)

Supplementary Table S2 : Contents in the supplementary file “supplementary_notebook2.zip”

File	Description
00_readme.txt	Direction to combine all files in two zip files
LICENSE	Licence information for the program code
mid.py	Python code for the integer decomposition algorithm
